# CRISPR library designer (CLD): software for multispecies design of single guide RNA libraries

**DOI:** 10.1186/s13059-016-0915-2

**Published:** 2016-03-24

**Authors:** Florian Heigwer, Tianzuo Zhan, Marco Breinig, Jan Winter, Dirk Brügemann, Svenja Leible, Michael Boutros

**Affiliations:** Division Signaling and Functional Genomics, German Cancer Research Center (DKFZ) and Heidelberg University, Im Neuenheimer Feld 580, Heidelberg, 69120 Germany; Department of Medicine II, University Hospital Mannheim, Medical Faculty Mannheim, Heidelberg University, Mannheim, Germany

**Keywords:** Bioinformatics, CRISPR/Cas9, sgRNA design, Pooled screens, Functional genomics

## Abstract

**Background:**

Genetic screens using CRISPR/Cas9 are a powerful method for the functional analysis of genomes.

**Results:**

Here we describe CRISPR library designer (CLD), an integrated bioinformatics application for the design of custom single guide RNA (sgRNA) libraries for all organisms with annotated genomes. CLD is suitable for the design of libraries using modified CRISPR enzymes and targeting non-coding regions. To demonstrate its utility, we perform a pooled screen for modulators of the TNF-related apoptosis inducing ligand (TRAIL) pathway using a custom library of 12,471 sgRNAs.

**Conclusion:**

CLD predicts a high fraction of functional sgRNAs and is publicly available at https://github.com/boutroslab/cld.

**Electronic supplementary material:**

The online version of this article (doi:10.1186/s13059-016-0915-2) contains supplementary material, which is available to authorized users.

## Background

The clustered regularly interspaced short palindromic repeats (CRISPR)-associated RNA-guided endonuclease Cas9 can be utilized in eukaryotic cells to introduce double-strand breaks at specific genomic sequences [1, 2]. There, the error-prone repair of double-strand breaks by non-homologous end joining results in nucleotide deletions and insertions, which can lead to gene inactivation. Further modifications of Cas9 have been developed, allowing for screening with activation (CRISPRa) or repression (CRISPRi) of target gene expression [3, 4]. Specificity of Cas9 knockout can be further enhanced by applying Cas9-nickase [5] or high fidelity Cas9 variants [6]. CRISPR/Cas9 technology works efficiently in many species [7–9] and the simplicity of this method allows screening in both cell culture [10–15] and whole organisms [8, 16]. In addition to the currently available human and murine genome-scale CRISPR libraries, there is a growing need for single guide RNA (sgRNA) libraries for custom gene sets, other organisms, and CRISPR type II endonucleases using alternative protospacer adjacent motif (PAM) sites such as Cpf1 [17]. While several web services for the gene-by-gene design of sgRNAs have been developed [18–22], integrated and flexible bioinformatics workflows for the design of custom sgRNA libraries are currently lacking.

Here, we present the CRISPR library designer (CLD) software, which implements an end-to-end design of custom sgRNA libraries targeting the genomes of many different species. We used this method to design a custom sgRNA library and performed a pooled screen to identify all known essential components of the TNF-related apoptosis-inducing ligand (TRAIL) pathway.

### Implementation

**Fig. 1:**
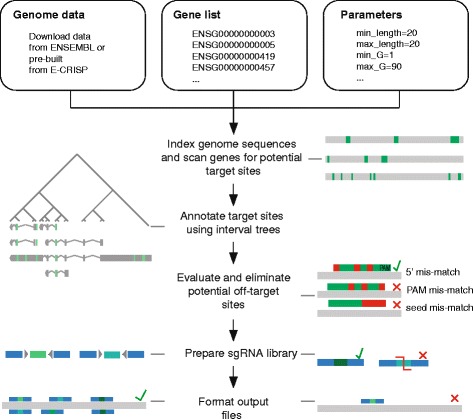
CRISPR library designer workflow. CLD is a command line tool, tailored for fast end-to-end design of sgRNA libraries. Its back-end steps are performed by the depicted algorithm: the genome data of the target organism, a gene list of interest, and a parameter file are needed as input files. Each gene sequence is then scanned for the presence of protospacer adjacent motifs (PAM). Valid target sites are evaluated for their annotation, sequence, and off-target characteristics and passed to the library formatting steps. There, target sites are tested for specific restriction sites and then flanked by cloning adapters. A user-defined minimum of best-annotated sgRNAs is selected for each gene. Genes with sgRNA coverage below the defined minimum are discarded. In the end, output files are generated, including a FASTA file containing ready-to-order oligonucleotide sequences

The CLD software package implements an end-to-end design of custom sgRNA libraries targeting the genomes of many different species. CLD automates all tasks for the generation of sgRNA libraries. It can design libraries of variable size ranging from a few hundred genes to genome-scale for all annotated genomes available from ENSEMBL [23]. CLD implements the following steps: (i) it downloads and reformats ENSEMBL databases, (ii) predicts and filters sgRNA target sites for a provided list of genes, and (iii) reports the results in a ‘ready-to-order’ library file containing nucleotide sequences for on-chip synthesis and subsequent cloning into target vectors. Figure 1 shows the schematic workflow of CLD. CLD requires three input files: the genome sequence, a parameter file, and a gene list. To ensure flexibility, genome sequence files can be downloaded either from ENSEMBL or as pre-calculated files from our website (http://www.dkfz.de/signaling/crispr-downloads/). In addition, the user supplies a parameter file to adapt design options, i.e., target site length, target region, or number of tolerable off-targets (Additional file 1: Table S1). These parameters enable the construction of custom libraries optimized for a broad spectrum of experimental applications. The third input file is a list of gene identifiers or genomic coordinates of regions to be targeted by sgRNAs (Additional file 2: Table S2). All input information can also be supplied via a user-friendly, graphical user interface (GUI). Target sites are identified using an algorithm which scans each gene for all possible sgRNA sites [24, 25]. A pattern-matching algorithm first indexes all PAM sequences by nucleotide positions and then searches this index to find matches. All potential sgRNAs (e.g., over 3000 for the human *MAPK1* gene) are then further filtered by user-defined criteria. In order to identify sgRNAs targeting specific, user-defined gene regions, CLD uses an interval tree containing all annotations of the genome. The user defines filtering parameters (e.g., coding regions, target length, exon targeting, start and stop codon targeting), which enable the design of libraries against protein-coding and non-coding genes or transcription start sites for CRISPRi and CRISPRa applications [4, 26] (see also Additional file 3: Figure S1). Target sequences of sgRNAs, which pass all filter criteria, are mapped to the genome of interest in order to identify up to 30 potential off-targets. Selection criteria for on- and off-targets can be custom defined including tolerance of mismatches at different nucleotide positions. On-target efficiency and frequency of off-target sites are then assessed by different scoring algorithms. First, potential off-target sites are identified using user defined alignment algorithms (bowtie, bowtie2, blastn-short) [27–29] and summarized in the specificity score. Then, the annotation score evaluates the target position of sgRNAs within the respective gene model. Finally, the nucleotide composition surrounding the target site is evaluated via the algorithms published by Doench et al. [30] and Xu et al. [31]. In addition, the user may supply a custom scoring algorithm to be integrated into CLD via a plug-in function (for details, see Methods, sgRNA scoring). Each sgRNA is ranked by specificity and annotation score. Additional ranking by Doench or Xu score can be selected. Next, target site sequences are processed to generate sgRNAs suitable for subsequent cloning steps, including addition of adapters and exclusion of specific restriction sites. Genes with coverage below a user-given threshold can be excluded. Finally, all data are reformatted into standardized file formats (GFF, FASTA, SAM; Additional file 4: Table S3). CLD can be run efficiently on desktop workstations (two to eight cores, 8 GB RAM) for smaller genomes or medium size gene lists. High-complexity genome-wide libraries have been calculated in <1 h on a 96 CPU server cluster.

## Results and discussion

### A pooled CRISPR/Cas9 screen for validation of CLD

**Fig. 2:**
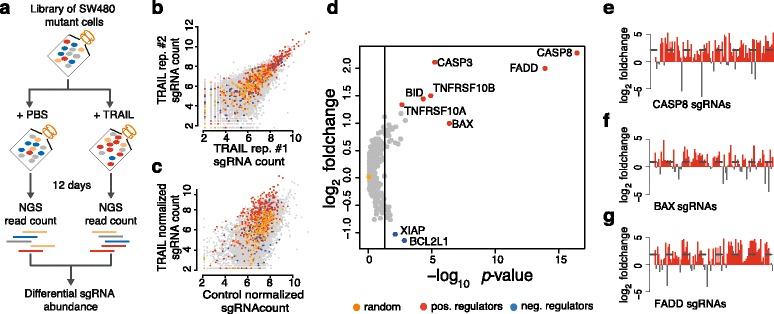
A pooled screen for functional validation of CLD. **a** The screening strategy in SW480 cells. In brief, a pool of mutant SW480 cells harboring 12,471 sgRNA designs against 408 genes was generated by lentiviral infection and antibiotic selection. Fourteen million cells per condition were then treated with PBS (control) or recombinant TRAIL (treatment) for a total of 12 days. Subsequently, the genomic DNA of the samples was extracted and sgRNA composition analyzed by next-generation sequencing (*NGS*). **b** Comparison of sgRNA sequence counts between two biological replicates demonstrates high reproducibility (Pearson correlation coefficient ~0.79). **c** Distribution of sgRNAs targeting positive pathway regulators (*CASP8*, *CASP3*, *FADD*, *BAX*, *BID*, *TNFRSF10A*, *TNFRSF10B*) in *red*, negative regulators (*XIAP*, *BCL2L1*) in *blue*, and random, non-targeting sgRNAs in *orange* between TRAIL (*y-axis*) and PBS (*x-axis*) treated cell populations. **d** Scatter plot showing relative enrichment of genes (*y-axis*) with their corresponding *p* value (*x-axis*). Positive regulators are plotted in *red*, negative regulators in *blue*, and random, non-targeting sgRNAs in *orange. P* values were calculated by Wilcoxon rank sum test between 30 sgRNAs of one gene and 200 random, non-targeting sgRNAs. Log_2_ fold change is calculated as median log_2_ ratio between normalized sgRNA count of TRAIL- over PBS-treated populations. The *vertical line* marks a *p* value of 0.05. **e**–**g** Median normalized fold change of all sgRNAs targeting three essential TRAIL pathway components. A total of 100 sgRNAs are depicted for each gene. Enriched sgRNAs are colored in *red*, depleted sgRNAs in *grey*. The *dashed line* represents the median fold change of all sgRNAs of the corresponding gene

To test the functionality of CLD, we designed a custom, ultra-complex library and tested it in a pooled screen in human cancer cells (raw data are provided in Additional file 5: Table S4, Additional file 6: Table S5, Additional file 7: Table S6, Additional file 8: Table S7). We chose to screen for modulators of TRAIL-induced apoptosis, as depletion of TRAIL pathway components results in distinct pro- or anti-apoptotic phenotypes [32]. Our custom library was composed of 12471 sgRNAs targeting 408 genes and including 200 non-targeting, randomly designed control sgRNAs (Additional file 3: Figure S3, Additional file 9: Table S8). We included positive (e.g., *CASP8*, *BAX*, *FADD*) and negative regulators (e.g., *BCL2L1*) of the TRAIL pathway, together with a large number of human protein kinases (Additional file 2: Table S2). Each gene was targeted by 30 different sgRNAs. The genes *BAX*, *CASP8*, and *FADD* served as positive controls and were targeted with approximately 100 sgRNAs. SW480 cells stably expressing Cas9 were transduced with the lentiviral sgRNA library. The pool of mutant cells was treated with either recombinant TRAIL or phosphate buffered saline (PBS) (Fig. 2a). The results of the screen showed that sgRNAs of specific genes were enriched or depleted upon TRAIL treatment, including known positive (e.g., *CASP8*, *BAX*, *FADD*) and negative regulators (e.g., *XIAP*, *BCL2L1*) (Fig. 2e, f; Additional file 3: Figure S2). Essential components of the pathway, such as *CASP8* or *FADD*, showed an average enrichment of approximately fourfold compared with non-targeting controls (*p* < 10^-3^, Wilcoxon rank sum test) (Fig. 2d). The receptors for TRAIL ligands (TNFRSF10A/DR4, TNFRSF10B/DR5), which are partially redundant [33], showed a weaker enrichment (Additional file 3: Figure S2a–c). sgRNAs against known negative regulators of the pathway are depleted with an average fold change of ~2 (Fig. 2b, c; Additional file 3: Figure S2e, f). Random, non-targeting sgRNAs showed a median log_2_ fold change around 0 (Fig. 2b–d; Additional file 3: Figure S3). The fold change of every sgRNA targeting *CASP8*, *FADD*, and *BAX* in the TRAIL treatment versus control group is shown in Fig. 2e–g. For these genes, more than 80 % of sgRNAs were enriched after exposure to TRAIL. For other hits, more than two-thirds of all sgRNAs showed an expected phenotype (Additional file 3: Figure S2), indicating that a high fraction of sgRNAs designed by CLD are indeed functional.

### Design parameters for sgRNA library

**Fig. 3:**
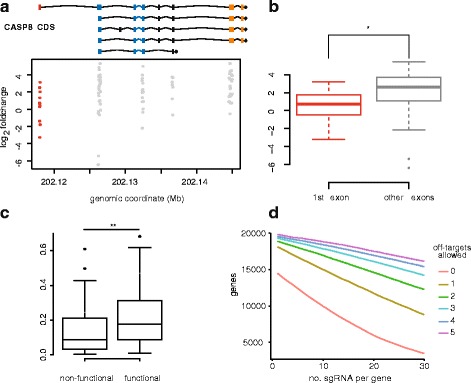
Impact of sgRNA features on library design. **a** Scatter plot showing log2 fold change of sgRNAs targeting *CASP8* relative to their exon location. The gene models of major transcripts of *CASP8* are depicted (ENST00000432109, ENST00000392258, ENST00000323492, ENST00000264275, ENST00000358485). **b** Box plots showing fold change of all sgRNAs targeting the first exon of *CASP8* compared with other targeted exons. sgRNAs against the first exon are less enriched (*p ≤ 0.05, two-sided *t*-test). **c** Comparison of sgRNA features between functional and non-functional sgRNAs. All sgRNAs of *BAX*, *FADD*, and *CASP8* were selected for analysis. Fold changes of individual sgRNAs of each gene were compared with the mean fold change of all sgRNAs of the respective genes. sgRNAs with a z-score >1 were grouped as functional and those with a z-score < −1 were grouped as non-functional. The on-target score by Doench et al. was calculated for both groups (*y-axis*) and are presented as box plots. Differences between groups were determined by a two-sided *t*-test. **d** Line graph showing interdependence between number of targetable human protein coding genes (*y-axis*), sgRNA coverage per gene (*x-axis*), and number of off-targets (colored lines). Off-targets are defined as genomic regions with at least 12 bases of homology to the protospacer of the on-target

Selection of sgRNAs with high on-target efficiency can reduce complexity of pooled libraries and facilitate screening. Understanding parameters that determine on-target efficiency is therefore essential for optimal library design. The results of our screen show that the functional impact of individual sgRNA is dependent on the exon being targeted. Using *CASP8* as a case example, we demonstrate that sgRNAs targeting the first exon are less enriched than those targeting other exons (Fig. 3a, b; *p* < 0.05, two-sided *t*-test). This can be explained by the gene model of *CASP8*: while the first exon is used by only few transcript variants, important functional domains are encoded by several exons [34]. In addition, nucleotide composition surrounding the PAM was found to determine on-target activity of sgRNAs [12, 13, 35]. The net effect of specific nucleotide features is summarized by two published algorithms [30, 31]. To assess the predictive power of these algorithms, we determined the efficiency of individual sgRNAs of *FADD*, *BAX*, and *CASP8* by comparing their fold change with the mean fold change of all sgRNAs of these genes. sgRNAs with a z-score >1 were classified as functional and those with a z-score < −1 were considered as non-functional. We then compared the two groups with regard to the scoring algorithm published by Doench et al. (Doench score) and Xu et al. (Xu score) by a paired *t*-test. We show that the Doench score is significantly different between the groups (Fig. 3c) whereas no difference was found for the Xu score (data not shown). Our data confirm that selecting sgRNAs with high Doench score may increase overall performance of sgRNA libraries. However, the number of sgRNAs with a high Doench score is limited (Additional file 3: Figure S4). In addition to on-target efficiency, the performance of sgRNA libraries is also determined by the specificity of selected sgRNAs. As shown in Fig. 3d, sgRNAs with no or few predicted off-targets are rare on a genome scale, as calculated using bowtie. Furthermore, the frequency of potential off-targets varies considerably between different organisms (Additional file 3: Figure S5).

### Limitations of in silico library design

Taken together, we provide experimental evidence that the algorithms implemented in CLD can reliably identify nucleotide sequence with high sgRNA activity. Using *CASP8* as a case example (Fig. 3a, b), we also confirm previous findings that sgRNAs targeting functional domains and common exons will more likely result in loss of function [11]. However, CLD’s ability to select appropriate target sites is dependent on the quality of genome annotations. While protein-coding transcripts of established model organisms and the human genome are well annotated, this is not the case for non-coding transcripts or the coding genomes of many other species, which might lead to a larger fraction of non-functional sgRNAs. In addition, off-targets are predicted by CLD based on sequence homology [27]. This limits off-target detection to sites with similar sequences (allowing up to three mismatches). Potential off-targets at sites with lower homology could remain undetected [36, 37]. Furthermore, the sensitivity for detecting off-target sites can vary depending on the algorithm used (bowtie versus blast). Thus, the rate of off-targets might be underestimated by CLD when bowtie is chosen as the alignment option. We also show that sgRNAs with high efficiency and/or specificity are rare on the genomic level and unequally distributed among genes, partly due to differences in gene-model length (Additional file 3: Figure S1). Furthermore, current knowledge about on-target efficiency is essentially derived from studies in murine and human cell lines and it is not known if they apply to other organisms as well. Therefore, design of comprehensive libraries will necessarily include sgRNAs with less predictable on-target efficiency and multiple off-targets. Furthermore, additional parameters that are unable to be computationally predicted can introduce variability in the performance of CRISPR/Cas9 screens. These include cell line-specific characteristics such as defective mismatch repair system, mutations/genetic variants [38], and differences in DNA accessibility to Cas9 [39, 40]. In addition, while screening for proteins essential for viral or toxin entry yields only few hits with highly penetrant phenotypes [10], perturbing complex signaling networks for drug resistance discovery will most likely reveal less distinct hits. These drawbacks can be partly overcome by screening with focused libraries with higher complexity, as was shown for small hairpin RNA screens [41]. While functional depletion of protein-coding genes by pooled CRISPR/Cas9 screens is highly efficient, targeting non-coding genes requires alternative strategies and library designs. A potential approach to dissect the function of enhancers is the use of saturating mutagenesis, i.e., targeting specific regions with as many sgRNAs as possible [42, 43].

## Conclusions

CLD provides all options to design highly customized sgRNA libraries to target both protein-coding genes and non-coding regions. The software is available to the community as an open source project.

## Methods

### Software infrastructure

CLD is implemented in Perl and is distributed as a stand-alone application together with the source code available at https://github.com/boutroslab/cld. It accepts 46 different input parameters to customize the library design (Additional file 1: Table S1) and outputs human and machine-readable files using commonly used sequence formats (FASTA, GFF; Additional file 4: Table S3). CLD has been optimized as a command line application for end-to-end design of sgRNA libraries in a single or paired design for use with various CRISPR/Cas9 systems. It also provides a graphical user interface for end-to-end design of libraries. The program has been combined with all its source packages and dependencies using the Perl-archiver (PAR) package. CLD was built and tested using the software versions as listed in Additional file 10: Table S9. CLD requires bowtie2 [28], bowtie [27], and blastn [29] for short-read mapping to be installed. CLD and the pre-built libraries can be downloaded from http://www.dkfz.de/signaling/crispr-downloads/. CLD is distributed as Unix binary bundle (https://github.com/boutroslab/cld).

### Scoring of sgRNAs

Target sites are identified by scanning each gene for the presence of specific PAMs. CLD creates a nucleotide index of each position containing a PAM. sgRNAs that harbor a TTTTT motif are excluded from further analysis as they would hinder RNA transcription [44]. When a match is found, three scores are calculated to evaluate the target site: annotation, specificity, and on-target efficiency score. The annotation score evaluates the efficiency of sgRNAs within the context of a gene model. Annotations such as exons, coding regions, or genes targeted by each sgRNA are found by searching all annotations at the target coordinates in an interval tree. This binary tree contains the coordinates and details of all annotations for the genome. The rooted binary tree structure can be searched efficiently (O = log(n)), minimizing computational resources. Trees are built and stored for every chromosome individually and are then retrieved from pre-computed binary files. The annotation score relies on general assumptions about positions at which sgRNAs should bind to efficiently alter the function of the respective gene. These positions are generally found in common transcripts and in coding exons, preferentially within close proximity of the transcription start site. The annotation score is calculated as follows: first it is set to 0; then all annotations overlapping the region at which the sgRNA under investigation binds are parsed; for each coding sequence and exon match, 5, divided by the number of the respective exons, is added; for every transcript it hits, 1 is added; for every start or stop codon hit, 1 is added; for every predicted CpG island, 1 is subtracted from the score. In summary, the annotation score enables CLD to sort sgRNA designs according to preferable target regions within a gene model. The specificity score is based on the assumption that specificity is determined by sequence homology of the 20 nucleotides preceding the PAM. Assuming that the first 5′ bases of the protospacer can possess ambivalent specificity [45], the user can exclude them from the specificity calculations. The remaining protospacer is mapped against the target genome using bowtie or blast in different adjustable modes (high or low sensitivity). For the highest sensitivity, up to three mismatches in the protospacer are allowed in the mapping. Furthermore, each mapped protospacer is required to be followed by a specific PAM. When all on- and off-targets of a single sgRNA are mapped, the specificity score is calculated. The score starts with a maximum of 100. If no off-targets exist, the score remains at 100. For each off-target, the number of homologous nucleotides of the off-target is subtracted from the score. This way, 20 is subtracted for a perfectly matching first off-target and another 10 is subtracted for a perfectly matching second off-target. For sgRNA libraries, all identified designs are sorted by best suitable annotation first, followed by target specificity and efficacy. sgRNA designs with the highest overall score are selected for inclusion in the library (see Additional file 11: Table S12 for details). On-target efficiency, determined by the nucleotide composition surrounding the NGG/NAG PAM site, is assessed by two published scoring algorithms: the Doench score [30] and the Xu score [31]. Optionally, an additional, user-provided score can be used to assess on-target activity. The custom score is only applied if provided to CLD as an external function. The custom score is limited to the assessment of a 30mer base sequence, starting at 24 nucleotides upstream of the PAM. The range of the score is limited to 0 to 1. The user may choose to further sort all sgRNA by an on-target score.

### Library design

We used CLD to design a custom sgRNA library consisting of 12,471 sgRNAs, of which 12,271 were directed against a total of 408 genes and 200 were random, non-targeting sgRNAs serving as controls. A list of selected genes can be found in Additional file 2: Table S2. All genes were covered with 30 sgRNAs per gene with the exception of *CASP8*, *FADD*, and *BAX*, which were covered with 100 sgRNAs. The library was designed by using the “end-to-end” functionality of CLD. Input files were the gene list from Additional file 2: Table S2 and the list of parameters in Additional file 12: Table S10. The parameters were set to score designs best if they target protein-coding regions of common exons outside of CpG islands. The source of all gene sequences and the basis of the off-target analysis was the human genome build GRCh37 ENSEMBL release 75. Targets were restricted to 23 nucleotides including PAM. The PAM was restricted to NGG and only ten off-targets were allowed, each with up to three possible mismatches in the first 16 5′ nucleotides. Off-targets were checked for each individual sgRNA by mapping the target site back to the genome. Before mapping, each sgRNA was trimmed for its last four nucleotides and mapped to the genome together with any possible PAM (AGG, TGG, CGG, GGG, AAG, TAG, CAG, GAG, etc.). Mismatches in the PAM were not allowed. A custom Perl script was used to generate random non-targeting sgRNA designs (Additional file 13: Supplementary file 1). These sgRNAs were designed by randomly generating 20,000 × 20mer oligonucleotides, which are mapped back to the human genome with loose parameters (ignore first four 5′-nucleotides, allow three further mismatches). Designs with successful alignment were excluded from this list. This resulted in a list of 2000 sgRNAs which did not map to the human genome, were compliant with the cloning strategy (no BbsI restriction sites), and were able to be expressed by DNA-dependent RNA polymerases (no TTTTT or GGGGG motifs). For library construction, each target site was trimmed by its first 5′ nucleotide and replaced by guanine. Adapters for cloning were added (Additional file 14: Table S11) and sequences with hindering restriction sites (GAAGAC, GTCTTC, GAATTC, CTTAAG, CAATTG, GTTAAC, CTCGAG, GAGCTC) were removed. Finally, designs were sorted hierarchically, in the following order: gene annotation, specificity, efficacy score. The 30 highest ranked designs were chosen for each gene. For *CASP8*, *FADD*, and *BAX*, designs from previously published libraries [12, 13] were included in addition to those designed by CLD. As a result, a set of uniquely named files is created in the output directory. A detailed description of each file can be found in Additional file 4: Table S3.

### Plasmid vectors

To clone Marie-U6-onchip, we modified the lentiviral vector pLKO.1 [46]. We first digested pLKO.1 with MefI (NEB) and then introduced gBlocks (Integrated DNA Technologies) encoding a FRT1-CMV-rtTA3-WPRE cassette using sequence- and ligation-independent multi-fragment cloning (InFusion cloning, Clontech). Then, the modified pLKO.1 was cut with AleI and KpnI, which removed the PGK-puromycin resistance cassette. Next, the U6 promotor and a truncated sgRNA cassette together with a mPGK-EM7-promotor-driven blasticidin-resistance cassette as well as a FRT3 site were introduced using sequence- and ligation-independent multi-fragment cloning (InFusion cloning, Clontech) of gBlocks (Integrated DNA Technologies). The blasticidin sequence and pLKO.1 were modified to remove all BbsI sites. Placing the blasticidin expression cassette under the control of an *Escherichia coli* promoter next to the sgRNA cassette allowed antibiotic selection of bacterial colonies containing correctly assembled vectors. The sgRNA cassette of Marie-U6-onchip vector contains only half of the sgRNA scaffold, preceded by two BbsI sites (referred to as on-chip design). The oligo library encodes the other half of the sgRNA cassette. Final lentiviral vectors were assembled by ligation of pooled oligos.

### Construction of sgRNA libraries

Oligonucleotide pools consisting of 12,471 different 99mers were ordered from CustomArrays Inc. (Bothell, WA, USA). The oligo sequences are provided in Additional file 14: Table S11 and Additional file 15: Supplementary file 2. We PCR amplified 1 ng of this oligo pool using primers onchip-F and onchip-R, Q5 Hot Start HF Polymerase (NEB) and the following PCR conditions: 98 °C for 10 s, 16 cycles of 98 °C for 10 s, 64 °C for 15 s, and 72 °C for 15 s, with a final extension at 72 °C for 2 min. The products of five PCR reactions were pooled and column purified with a NucleoSpin Gel and PCR clean-up kit (Machery-Nagel), followed by restriction digestion with Fast Digest BbsI (Thermo Fisher) for at least 12 h at 37 °C and another round of column purification. After every purification step, correct oligo size was confirmed using DNA High Sensitivity Assay on a BioAnalyzer 2100 (Agilent). The backbone vector Marie-U6-onchip was digested with Fast Digest BbsI and dephoshorylated with Fast Alkaline Phosphatase (NEB) for 16 h and loaded on a 0.8 % agarose gel to confirm successful digestion. The vector was then excised from the gel and purified using a NucleoSpin Gel and PCR clean-up kit (Machery-Nagel). Concentrations of digested backbone vector and oligo pools were determined using a Qubit dsDNA HS Assay (Life Technologies). We ligated 10 ng of backbone vector and 340 ng of oligonucleotides per reaction using T4 DNA Ligase (NEB) for 16 h at 16 °C. Five reactions were combined and cleaned using a Qiaquick PCR purification Kit (Qiagen) and eluted into nuclease-free water. The concentration of ligated vector was determined by Qubit dsDNA HS Assay (Life Technologies). A total of ten electroporations were performed according to the manufacturer’s protocol using 1 ng of ligated vector and 25 ul of DH10beta *E. coli* Electrocompetent Cells (NEB). Each electroporation reaction was then plated onto two 15-cm diameter agar plates containing Luria broth medium (Life Technologies) and 100 μg/ml carbenicillin. After overnight incubation at 37 °C, bacterial colonies equaling 500-fold library complexity were scraped off all plates, pooled, and purified with a Plasmid Maxi Kit (Qiagen).

### Cell culture and generation of Cas9-expressing cells

The colorectal cancer cell line SW480 is highly sensitive to TRAIL (Additional file 3: Figure S8) and was previously used to study TRAIL signaling [32, 47]. SW480 cells were maintained in RPMI medium (Invitrogen) containing 10 % fetal calf serum (Biochrom). HEK 293T cells were kept in high glucose Dulbecco's modified Eagle's medium (DMEM) (Invitrogen) supplemented with 10 % fetal calf serum (Biochrom). Both cell lines were obtained from ATCC. Authentication of genotype by SNP profiling (Multiplexion) was performed on all cell lines and regular tests confirmed the absence of mycoplasma infection. Stable Cas9-GFP expression was achieved by using piggybac transposase/transposon technology and subsequent selection of green fluorescent protein (GFP)-positive cells by fluorescence-activated cell sorting (FACS) (T.Z., M.Br. unpublished).

### Lentivirus production and infection

For lentivirus production, HEK293T cells were seeded into two T225 flasks (Nunc) at a density of 6 × 10^5^ cells/ml (30 ml per flask) and incubated for 24 h, after which a confluence of 80 % is reached. We added 90 μg of sgRNA library, 60 μg psPAX2, and 20 μg of pMDM2 (both from Addgene) to a total of 6 ml RPMI and 300 μl of TransIT (Mirus) was added to 5.7 ml of RMPI. After 10 min, both solutions were mixed and incubated for another 30 min before adding to both flasks (6 ml/flask). After 24 h, medium was exchanged to DMEM containing 10 % fetal calf serum and 1 U/ml DNAseI (Thermo Fisher). Viral supernatant was harvested 48 h after transfection and stored at −80 °C until use. For determining multiplicity of infection (MOI), lentiviral supernatant was generated using the GFP-expressing vector pLKO-G3 (Addgene) under the same conditions and used as a surrogate.

### Generation of mutant cell libraries and screening

To determine the MOI, 10^5^ SW480 cells were seeded into each well of a 12-well plate (Greiner). While in suspension, cells were infected with increasing volumes of the pLKO-G3 derived lentivirus in the presence of 5 μg/ml polybrene (Merck Millipore). Cells were detached 72 h post-infection and resuspended in MACS buffer (PBS with 1 % fetal calf serum and 2 μM EDTA). The percentage of GFP positive cells for each volume of lentiviral supernatant was determined by FACS analysis on a FACS Canto (BD). For generation of mutant libraries, 4 × 10^7^ SW480 cells were infected with the sgRNA lentiviral library equivalent to an MOI of 0.2–0.3 (1000-fold library complexity) in the presence of 5 μg/ml polybrene (Merck Millipore). After 72 h, cells were detached and reseeded onto new flasks in the presence of 4 μg/ml blasticidin (Life Technologies). Antibiotic selection was terminated after 72 h and cells were allowed to recover for another 5 days. Cells were then harvested and either stored in liquid nitrogen or directly used for screening. For screening, 1.4 × 10^7^ cells were used per replicate, equivalent to a >1000-fold library complexity. For each condition, two replicates were used. After 24 h, cells were treated with 100 ng/ml of water-soluble SuperKillerTRAIL (Enzo Life Sciences) or PBS for 24 h. The medium was then replaced and the cells were allowed to recover for 5 days. Thereafter, SuperKillerTRAIL was added for another 24 h, followed by medium change and a recovery phase of 5 days. After a total of 12 days, at least 1.4 × 10^7^ cells were harvested from each replicate.

### Genomic DNA isolation and library preparation for Illumina sequencing

Genomic DNA from cell pellets containing 1.4 × 10^7^ cells were extracted using the DNAeasy Blood and Tissue Kit (Qiagen) according to the manufacturer’s protocol. For amplification of the sgRNA-containing regions, a total of 25 PCR reactions were performed using 1 μg genomic DNA per reaction as input, Q5 Hot Start HF polymerase (NEB), and primers SEQ-F1 and SEQ-R1 with the following conditions: 98 °C for 2 min, 25 cycles of 98 °C for 10 s, 62 °C for 15 s, and 72 °C for 30 s, with a final extension at 72 °C for 2 min. The PCR product was cleaned using a QIAquick PCR purification Kit and eluted into nuclease-free water. The DNA concentration of the eluate was determined using Qubit HS DNA Assay. The purified PCR product (5 ng) was used for enrichment PCR with Q5 Hot Start HF polymerase (NEB), primers SEQ-F2 and SEQ-R2, and the following PCR conditions: 98 °C for 2 min, 15 cycles of 98 °C for 10 s, 72 °C for 15 s, and 72 °C for 30 s, with a final extension at 72 °C for 2 min. The PCR product was purified with Agencourt AMPure XP beads at a product-to-beads ratio of 1:1.2. The purified libraries were controlled for correct size using DNA High Sensitivity Assay on a BioAnalyzer 2100 (Agilent) and then sequenced on a MiSeq (Illumina) by 100-bp single-end sequencing and addition of 20 % PhiX Control v3 (Illumina) at a concentration of 8 pM. Two MiSeq runs were performed each containing one replicate of the TRAIL- and PBS-treated conditions.

### Illumina sequencing of plasmid libraries

For determining library coverage, 750 ng of the purified plasmid library was amplified using primers SEQ-F2 and SEQ-R2, Phusion High Fidelity Polymerase (Biozym), and the following PCR conditions: 98 °C for 2 min, 15 cycles of 98 °C for 10 s, 72 °C for 15 s, and 72 °C for 30 s, with a final extension at 72 °C for 2 min. The PCR product was purified with a Qiaquick PCR purification kit (Qiagen). The purified libraries were controlled for correct size using DNA High Sensitivity Assay on a BioAnalyzer 2100 (Agilent) and then sequenced on a HiSeq 2500 (Illumina) by 100-bp paired-end sequencing and addition of 20 % PhiX Control v3 (Illumina) at a concentration of 8 pM. All primer sequences can be found in Additional file 14: Table S11.

### Data processing and analysis

Reads reported by the MiSeq analysis were quality checked using FASTQC and analyzed FASTQ data using a custom Perl script, which can be found in Additional file 16: Supplementary file 3. The sequencing reads were checked and trimmed for the adapters, which were added in silico before on-chip synthesis of the library. These adapters are part of the expression cassette resulting in the following required pattern: ACCG(.{20})T{2,4}AGAGC (Perl-regular expression). The target site (all nucleotides in the parentheses of the pattern) is saved in a new variable and in the next step mapped back to the original library. As a result we obtained sgRNA count tables for each sample: negative control 1 (PBS1), negative control 2 (PBS2), TRAIL treatment 1 (TRAIL1), and TRAIL treatment 2 (TRAIL2) (Additional file 5: Table S4, Additional file 6: Table S5, Additional file 7: Table S6, Additional file 8: Table S7, respectively). The raw read counts were processed using an algorithm implemented in R. The source code is attached in Additional file 17: Supplementary file 4. In short, raw counts were collected and divided by the respective sample median for normalization and the log_2_ fold change was calculated as the log_2_-ratio between the mean read count in treated samples and the mean read count per sgRNA of the control samples (Fig. 2e–g; Additional file 3: Figure S2,S3). Read counts in Fig. 2b, c were normalized. The median fold change of the random controls was set to zero by subtracting it from every fold change in the dataset. This corrected for the general loss of coverage during the TRAIL treatment. For testing sample sgRNAs against random controls, a Wilcoxon rank sum test was performed under the null hypothesis that the true shift of means is larger than zero. The statistical significance of differences in parameters between enriched and non-enriched sgRNAs in sample genes was assessed using a two-sided Student’s *t*-test as implemented in R with default parameters under the null hypothesis that the true difference in means is larger than zero. All analysis scripts can be found in Additional file 17: Supplementary file S4.

## Availability of data and material

Raw sequencing reads have been deposited at the Sequence Read Archive (SRA; project ID SRP070542): PBS-treated pool 1, SRR3178382; PBS-treated pool 2, SRR3178383; TRAIL-treated pool 1, SRR3178384; TRAIL-treated pool 2, SRR3178385.

A release version of CLD can be found at http://dx.doi.org/10.5281/zenodo.46772. The software presented here is licenced under GPLv2. All other scripts and software to reproduce the results can be found in the supplemental material.

## Ethics approval

No ethics approval was required for this study.

## Additional files

Additional file 1:
**Table S1.** Detailed list and description of available parameters used in CLD. (XLSX 13 kb) Additional file 2:
**Table S2.** ENSEMBL ID to gene symbol mapping. (XLSX 58 kb) Additional file 3: Supplementary Figures S1-S6, showing additional experimental data or design aspects of CLD. (PDF 214 kb) Additional file 4:
**Table S3.** File formats, as output of CLD. (XLSX 39 kb) Additional file 5:
**Table S4.** Counts after processing the raw read files with Additional file 17: Supplementary file 4 for PBS1. (XLSX 458 kb) Additional file 6:
**Table S5.** Counts after processing the raw read files with Additional file 17: Supplementary file 4 for PBS2. (XLSX 461 kb) Additional file 7:
**Table S6.** Counts after processing the raw read files with Additional file 17: Supplementary file 4 for TRAIL1. (XLSX 444 kb) Additional file 8:
**Table S7.** Counts after processing the raw read files with Additional file 17: Supplementary file 4 for TRAIL2. (XLSX 414 kb) Additional file 9:
**Table S8.** Re-annonation of the sgRNA library targeting the TRAIL pathway. (XLSX 1883 kb) Additional file 10:
**Table S9.** Software used in this study. (XLSX 39 kb) Additional file 11:
**Table S12.** Detailed description of CLD’s scoring scheme. (DOCX 102 kb) Additional file 12:
**Table S10.** Detailed list of available parameters used in CLD. (XLSX 41 kb) Additional file 13:
**Supplementary file 1.** Perl script generating random sgRNA target sites. (PL 1 kb) Additional file 14:
**Table S11.** Sequences of primers and oligos used for cloning of sgRNA libraries and sequencing. (XLSX 9 kb) Additional file 15:
**Supplementary file 2.** TRAIL targeting high complexity oligo library. (FASTA 1564 kb) Additional file 16:
**Supplementary file 3.** Perl script used for raw read procession and sgRNA read count. (PL 15 kb) Additional file 17:
**Supplementary file 4.** Analysis scripts bundled as R markdown. (RMD 27 kb)

## Abbreviations

CLD: CRISPR library designer
CRISPR: clustered regularly interspaced short palindromic repeats
CRISPRa: CRISPR activator variant
CRISPRi: CRISPR repressor variant
DMEM: Dulbecco's modified Eagle's medium
FACS: fluorescence-activated cell sorting
GFP: green fluorescent protein
MOI: multiplicity of infection
PAM: protospacer adjacent motif
PBS: phosphate buffered saline
sgRNA: single guide RNA
TRAIL: TNF-related apoptosis-inducing ligand
