# Supplementary material for: CRISPR library designer (CLD): software for multispecies design of single guide RNA libraries
Source: Genome Biol. 2016 Mar 24;17:55. doi: 10.1186/s13059-016-0915-2 (PMC4807595; doi:10.1186/s13059-016-0915-2)
Supplement: Additional file 3: — Supplementary Figures S1-S6, showing additional experimental data or design aspects of CLD. (PDF 214 kb) [file 13059_2016_915_MOESM3_ESM.pdf]

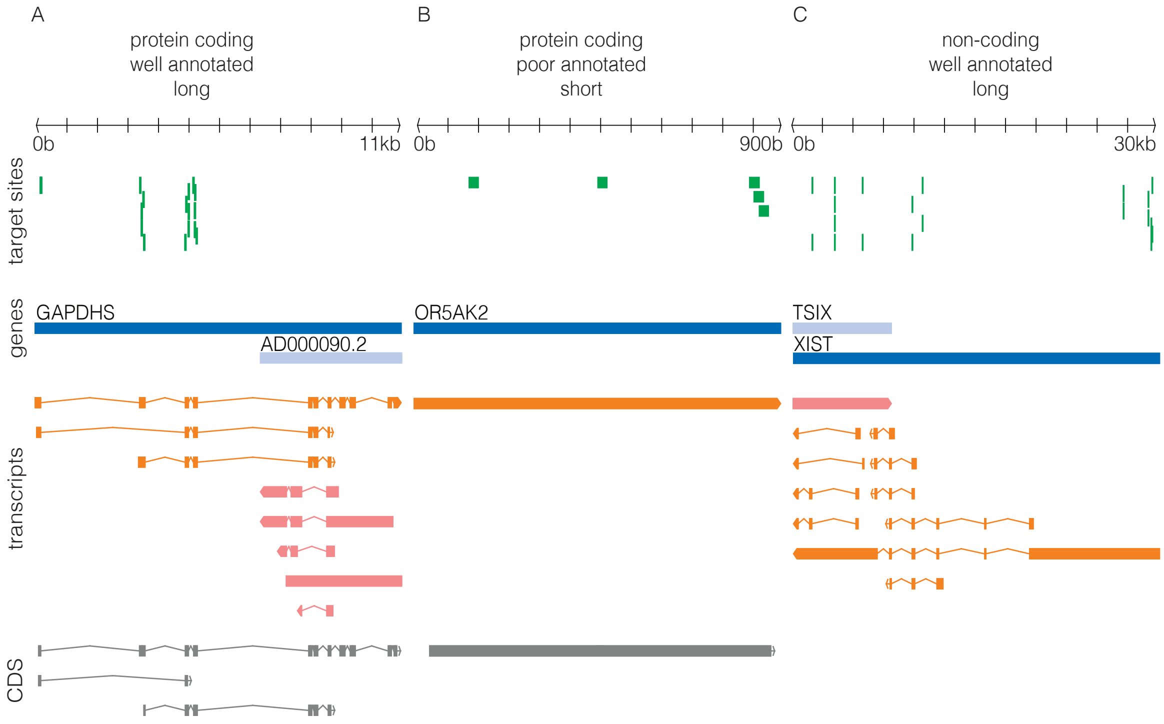

Supplementary Figure 1: The influence of gene models on design criteria

The three human genes GAPDHS, OR5AK2 and XIST were subject to sgRNA design. (A) GAPDHS is a case example for a well-annotated gene. It overlaps with the gene AD000090.2 in anti-sense direction. In order to prevent off-target effects, sgRNAs targeting overlapping gene regions are excluded. (B) OR5AK2 is one of the shortest protein coding genes in the human genome. It has a very short coding sequence and no introns. The number of sgRNAs that target OR5AK2 is limited as a consequence of its small size. (C) XIST is an example for a well-studied long non-coding RNA. Although this lncRNA has no protein-coding region, it features a structured gene model amenable to sgRNA targeting.

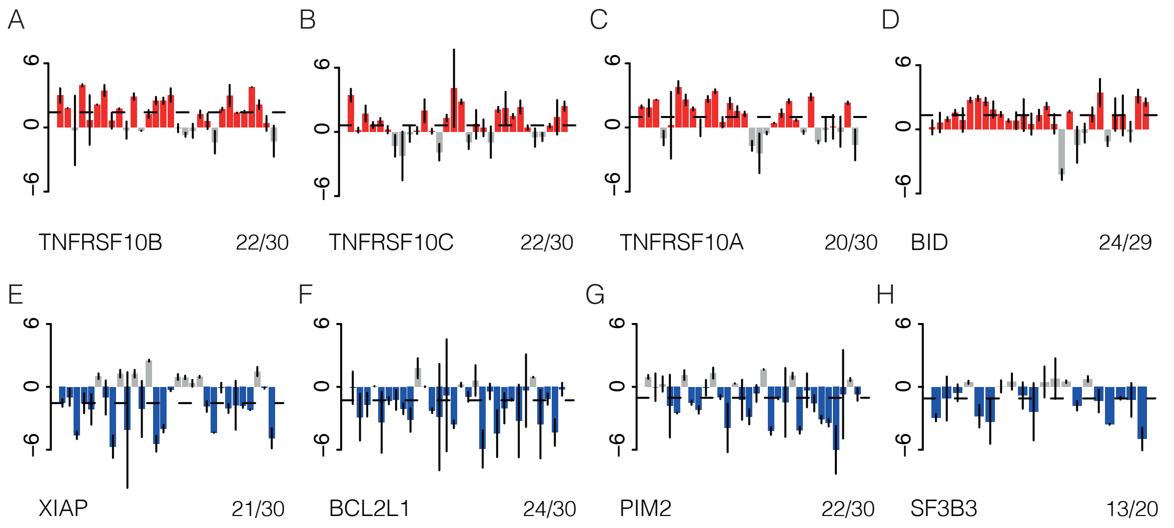

Supplementary Figure 2: Normalized fold-change of hits with intermediate phenotypes

Bar chart showing median normalized fold-changes of all sgRNA for selected enriched (A-D) and depleted (E-H) genes. All genes were identified as significant hits in this screen (p-value < 0.05, two sided t-test). Enriched sgRNAs are colored in red and depleted sgRNAs are colored in blue. A dashed line demarks the median fold-change of all sgRNA. Error bars show standard deviation between two biological replicates.

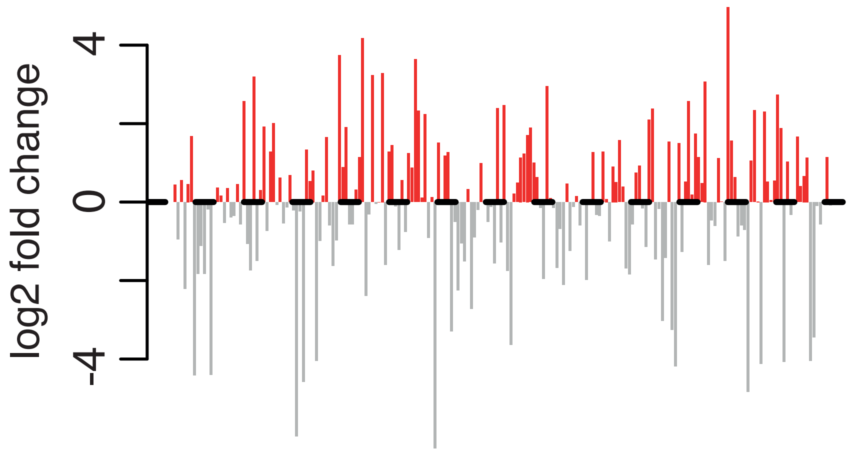

Supplementary Figure 3: Normalized fold change of random, non-targeting sgRNAs

Bar chart showing median normalized fold changes of all random, non-targeting sgRNAs of two replicates. Enriched sgRNAs are colored in red and depleted sgRNAs are colored in grey. Random, non-targeting sgRNAs showed a median fold change around 0, but with a considerable variance between individual sgRNAs.

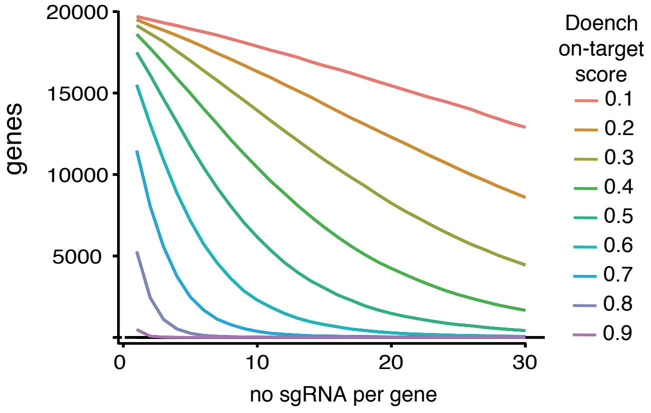

Supplementary Figure 4: Influence of Doench on-target score on library complexity

Line graph showing interdependence between number of targetable human protein coding genes (y-axis), sgRNA coverage per gene (x-axis) and different Doench on-target score thresholds (coloured lines).

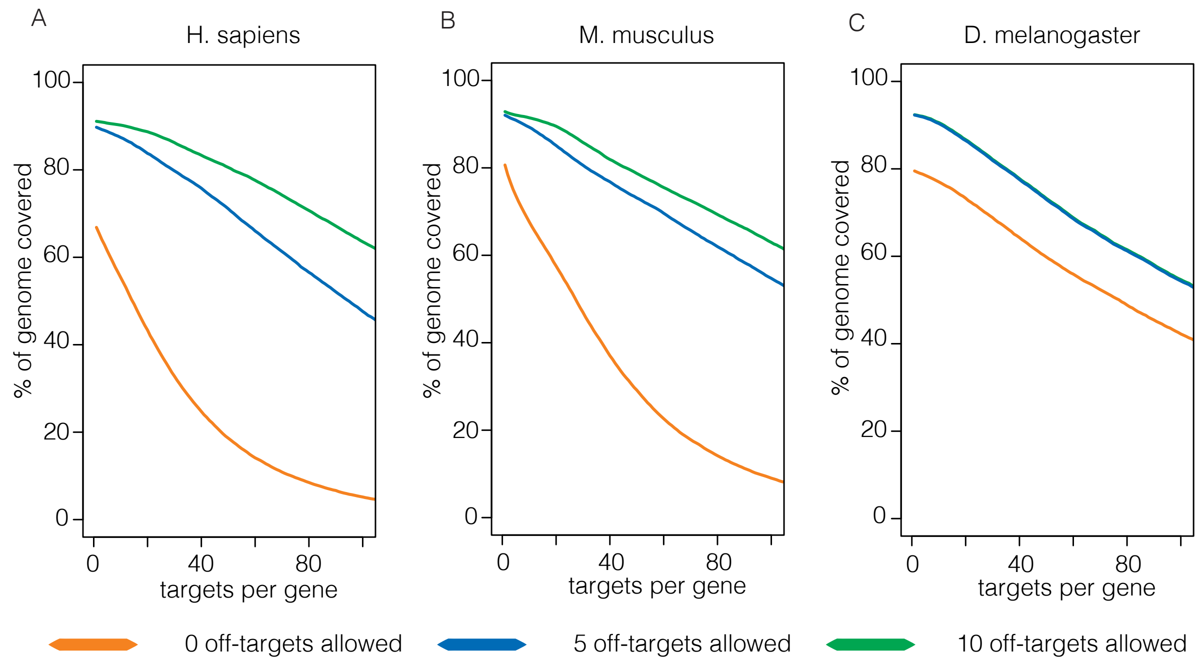

Supplementary Figure 5: Influence of off-target tolerance on library complexity

Line graphs showing percentage of genome that can be targeted (y-axis) relative to the number of sgRNA per gene (y-axis) and off-target tolerance (coloured). Analysis was performed for the genome of (A) *Homo sapiens*, (B) *Mus musculus* and (C) *Drosophila melanogaster*. The percentage of genome (consisting of protein-coding genes) that can be targeted by multiple sgRNAs decreases when a minimal number of off-targets is allowed.

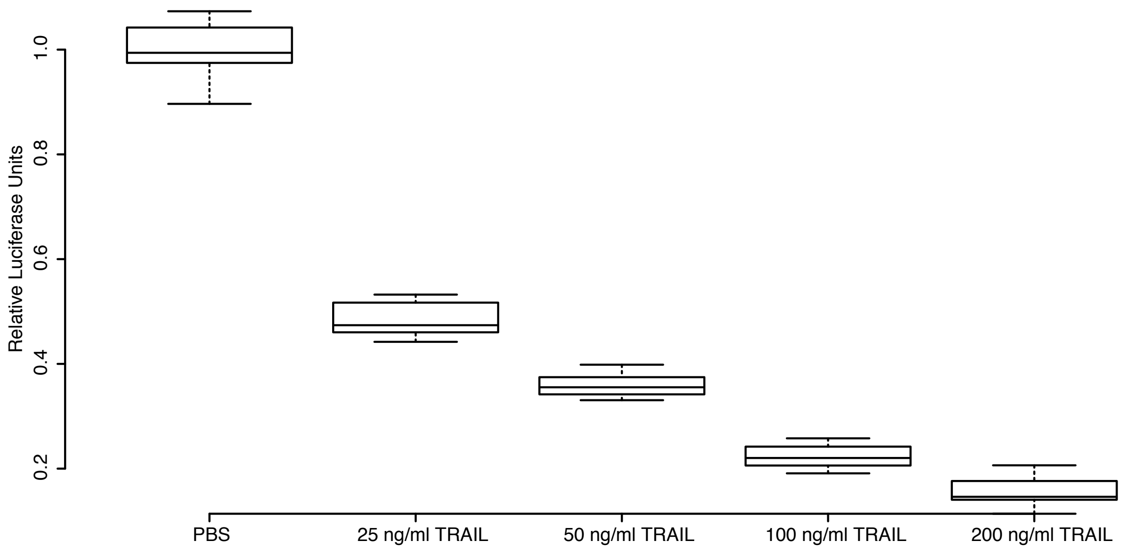

Supplementary Figure 6: Effect of TRAIL treatment on viability of SW480 cells.

SW480 cells (12,000 per well) were plated in 96-well plates 24h before treatment. The cells were treated with the indicated concentrations of recombinant TRAIL. The number of viable cells was determined 72h after the initiation of treatment using Cell Titer Glo (CTG) luminescent cell viability reagent (Promega). CTG was diluted 1:5 in PBS and added to each well after removal of cell culture medium. Luminescence was measured with a Mithras LB 940 (Berthold). Box plot show viability of cells under different TRAIL concentrations, normalized to PBS treated controls. Data for each condition was collected from 10 technical replicates and two biological replicates.

References

1. Wang, T., Wei, J. J., Sabatini, D. M. & Lander, E. S. Genetic screens in human cells using the CRISPR-Cas9 system. *Science* **343,** 80–4 (2014).

2. Shalem, O., Sanjana, N. E. & Zhang, F. High-throughput functional genomics using CRISPR-Cas9. *Nat. Rev. Genet.* **16,** 299–311 (2015).

3. Ren, X. *et al.* Optimized gene editing technology for Drosophila melanogaster using germ line-specific Cas9. *Proc. Natl. Acad. Sci. U. S. A.* (2013). doi:10.1073/pnas.1318481110

4. Doench, J. G. *et al.* Rational design of highly active sgRNAs for CRISPR-Cas9–mediated gene inactivation. *Nat. Biotechnol.* **32,** 1262–7 (2014).

5. Li, W. *et al.* MAGeCK enables robust identification of essential genes from genome-scale CRISPR/Cas9 knockout screens. *Genome Biol.* **15,** 554 (2014).
