# Supplementary material for: CRISPR library designer (CLD): software for multispecies design of single guide RNA libraries
Source: Genome Biol. 2016 Mar 24;17:55. doi: 10.1186/s13059-016-0915-2 (PMC4807595; doi:10.1186/s13059-016-0915-2)
Supplement: Additional file 11: — Table S12. Detailed description of CLD’s scoring scheme. (DOCX 102 kb) [file 13059_2016_915_MOESM11_ESM.docx]

| Score | Calculation |
| --- | --- |
| Specificity | The specificity score evaluates the specificity of an sgRNA by measuring off-target abundance.  The score is based on the assumption that specificity is determined by sequence homology of the 20 nucleotides preceding the protospacer adjacency motif (PAM). Assuming that the first 5’ bases of the protospacer can possess ambivalent specificity, the user can exclude it from the specificity calculations. The remaining protospacer is mapped against the target genome using bowtie in different adjustable modes (high or low sensitivity). For the highest sensitivity, up to three mismatches in the protospacer are allowed in the mapping. Furthermore each mapped protospacer is required to be followed by a specific PAM (NAG/NGG). When all on- and off-targets of a single sgRNA are mapped, the specificity score is calculated. The score starts with a maximum of 100. If no off-targets exist, the score remains at 100. For each off-target, the number of homologous nucleotides of the off-target divided by the off-target count is subtracted from the score. For example, if a perfectly matching off-target exists, 20 is subtracted. If a second perfectly matching off-target exists, another 10 is subtracted. |
| Annotation | The annotation score evaluates the efficiency of an sgRNA with regard to its binding position within the respective gene model.  This score is based on general assumptions that binding of sgRNAs at different positions within a gene model will affect the gene function differently. Preferable positions for sgRNA binding to achieve altered gene functions are located in common transcripts and in early coding exons. Therefore the score is calculated the following way: First, it is set to 0. Then all annotations overlapping the region, where the sgRNA binds, are parsed. For each coding sequence and exon 5, divided by the number of the respective exons, is added. For every gene that is hit, 1 is added. For every start or stop codon hit, 1 is added. For every computationally predicted CpG island, 1 is subtracted from the score. In the current version of CLD, it is possible to provide a weight to every aspect of this score, e.g. to give much more weight on targeting coding exons, rather than UTR’s. |
| Extra | CLD users can apply any custom perl algorithm that relies on sequence characteristics of the 30 nucleotides surrounding the protospacer target site (see also Doench et. al). The scoring function needs to have a 30-character string as input and a numeric characters output. |
